# Female cats, but not males, adjust responsiveness to arousal in the voice of kittens

**DOI:** 10.1186/s12862-016-0718-9

**Published:** 2016-08-12

**Authors:** Wiebke S. Konerding, Elke Zimmermann, Eva Bleich, Hans-Jürgen Hedrich, Marina Scheumann

**Affiliations:** 1 Institute of AudioNeuroTechnology and Department of Experimental Otology, ENT Clinics, Hannover Medical School, Stadtfelddamm 34, 30625 Hannover, Germany; 2 Institute of Zoology, University of Veterinary Medicine Hannover, Bünteweg 17, 30559 Hannover, Germany; 3 Institute for Laboratory Animal Science and Central Animal Facility, Hannover Medical School, Carl-Neuberg-Straße 1, 30625 Hannover, Germany

**Keywords:** Arousal, Infant cry, Isolation call, Sex-specific responsiveness, Domestic cat

## Abstract

**Background:**

The infant cry is the most important communicative tool to elicit adaptive parental behaviour. Sex-specific adaptation, linked to parental investment, may have evolutionary shaped the responsiveness to changes in the voice of the infant cries. The emotional content of infant cries may trigger distinctive responsiveness either based on their general arousing properties, being part of a general affect encoding rule, or based on affective perception, linked to parental investment, differing between species. To address this question, we performed playback experiments using infant isolation calls in a species without paternal care, the domestic cat. We used kitten calls recorded in isolation contexts inducing either Low arousal (i.e., isolation only) or High arousal (i.e., additional handling), leading to respective differences in escape response of the kittens. We predicted that only females respond differently to playbacks of Low versus High arousal kitten isolation calls, based on sex-differences in parental investment.

**Results:**

Findings showed sex-specific responsiveness of adult cats listening to kitten isolation calls of different arousal conditions, with only females responding faster towards calls of the High versus the Low arousal condition. Breeding experience of females did not affect the result. Furthermore, female responsiveness correlated with acoustic parameters related to spectral characteristics of the fundamental frequency (F0): Females responded faster to kitten calls with lower F0 at call onset, lower minimum F0 and a steeper slope of the F0.

**Conclusions:**

Our study revealed sex-specific differences in the responsiveness to kitten isolation calls of different arousal conditions independent of female breeding experience. The findings indicated that features of F0 are important to convey the arousal state of an infant. Taken together, the results suggest that differences in parental investment evolutionary shaped responsiveness (auditory sensitivity/ motivation) to infant calls in a sex-specific manner in the domestic cat.

**Electronic supplementary material:**

The online version of this article (doi:10.1186/s12862-016-0718-9) contains supplementary material, which is available to authorized users.

## Background

The infant cry is an important vocalisation as it triggers adaptive care-giving behaviours which are critical for the survival of new-born infants [1, 2]. The high salience of the infant cry in comparison to other vocalisations [3] is suggested to have evolved in mutual adaptation of acoustic parameters of the cry and the care-givers’ perceptual properties [4–6]. Besides its function in eliciting attentiveness to promote care-giving behaviour (“fight for priority” [4]), the infant cry can reliably convey the current need for support (e.g., defence or nutrition) of an isolated infant. Related to the infant’s need for support, the acoustic properties of the infant cry differ according to the arousal of the sender both in humans and nonhuman mammals (e.g., human: [2, 7]; nonhuman Primates: [8]; Artiodactyla: [9]; Proboscidea: [10]; Carnivora: [11, 12]; Chiroptera: [13]).

While several studies revealed that infant isolation calls trigger maternal care-giving behaviour (e.g., Artiodactyla: [14, 15]; Carnivora: [16, 17]; Primates: [18, 19]; Chiroptera: [20, 21]; Rodentia: [22, 23]), there is only limited knowledge on nonhuman male responsiveness to infant calls. Only in mice, common marmosets and deer has responsiveness towards infant calls been compared across sexes (mice: [4, 24, 25]; common marmoset: [26–28]; deer: [29]). In common marmosets all group members (i.e., males and females) are involved in infant-care (e.g., [30, 31]) and playback studies showed that fathers and mothers do not differ in their responsiveness to infant vocalisations. Furthermore, also inexperienced (i.e., naïve) males showed a strong preference for infant versus adult vocalisations [27]. Also for laboratory mice, playback studies showed that experienced fathers and mothers prefer infant vocalisations over control sounds [24, 25, 32]. Nevertheless, gradual sex differences exist in the necessary sensitisation time with pups, which may differ between mice strains. Whereas NMRI female mice show a preference for isolation calls (i.e., 50 kHz tones) already after co-caring for pups for 5 days [32], males need a co-caring period of at least 10 days [33]. These sex-differences are not apparent in ICR mice [25]. In contrast to the above mentioned studies, mule deer show sex-specific responsiveness towards fawn isolation calls [29]. While females approached a loud-speaker playing back fawn isolation calls, male deer did not [29]. Taken together, these studies revealed that in species with biparental care, sex-differences in response to infant cries are subtle, if present at all, whereas in species without paternal care, males seem to be indifferent to infant calls.

In humans, men and women, independent of parental status, are able to recognise the emotional content of infant vocalisations (e.g., [34–38]). The ability of nonhuman mothers to adjust responsiveness to the emotional content of the infant cry has only been shown in two vertebrate species, the sow [39] and the black caiman [40], which are both species with a maternal breeding system. No study has analysed whether the ability to adjust responsiveness to the voice of an infant is generalised to non-caretakers (e.g., males and naïve females). Thus, the question arises, whether the emotional content of infant cries can trigger distinctive responsiveness based on their general arousing properties, being part of a general affect encoding rule, or whether affective perception is linked to parental investment.

To fill this gap, we investigated the responsiveness of male and female domestic cats, a species without paternal care, towards infant vocalisations of different arousal conditions. Cats are an important animal model in human hearing research due to similarities in their auditory system (e.g., [41, 42]) and their well-described vocal repertoire [43–48]. In cats, litters from different females may be reared in the same nest but males are not tolerated around the nest site [49]. Kittens produce mammalian typical infant cries when isolated from their mother [11, 44–48, 50], that evoke maternal behaviour [17]. In a previous publication [11] we could already show that isolation calls recorded in contexts of high and low arousal differ in temporal and spectral parameters related to the fundamental frequency (F0). The Low arousal calls were recorded in a context of spatial separation, in which a kitten was left undisturbed and did not show signs of elevated arousal (i.e., moved around slowly, searching and calling). The High arousal calls were elicited by additionally manipulating the kitten (i.e., grasping and/ or turning over on the back), which was assumed to induce a higher level of urgency/ arousal, as kittens struggled with their legs and tried to turn around. However, to date it is unclear whether the related acoustic differences are biologically meaningful and can be decoded by adult male and female cats. In the present study we conducted playback experiments using kitten isolation calls of Low and High arousal. We predicted that females, but not males, adjust their responsiveness to the arousal state of kittens conveyed by infant vocalisations and thus will respond faster to High than Low arousal calls.

## Methods

### Subjects

Playback experiments were performed on 17 adult cats (9 males, 8 females) aged 1–8 years (mean_m_ = 2.4, mean_f_ = 3.6). All subjects were not neutered/ castrated and originated from and were kept at the breeding facility of the Central Animal Facility of the Hannover Medical School. Half of the females (*N* = 4) had already raised offspring and thus were defined as experienced. None of the females was pregnant or lactating during the time of the study. Adult cats lived in same-sex groups of 2–5 individuals, with changing composition based on breeding schedules. The cats were kept indoors in a controlled environment (light–dark cycle 12:12, 22 **±** 2 °C, 55 ± 10 % humidity). The rooms (12.5 m^2^ - 20.6 m^2^, height: 2.6 m) were enriched with wooden boxes, tables and shelves, plastic toys and bars for scratching. As an additional heat source, each room was equipped with an infrared lamp. The cats were fed daily with tinned (Whiskas® tins, Mars GmbH, Verden, Germany) and dry cat food (SDS Pet Food, Special Diets Services, Witham, Essex, UK) and were provided with water *ad libitum*.

### Recordings of playback stimuli

We recorded kitten isolation calls in two behaviourally defined conditions, of Low and High arousal (for details see [11]). The calls were recorded from 16 kittens (8 males, 8 females; 9 to 11 days of age) via a Sennheiser microphone (ME 67, Sennheiser, Wedemark, Germany: 40–20,000 Hz ± 2.5 dB) and Marantz recorder (PMD 660, Marantz, D&M Holdings Inc., Mahwah, NJ, USA; sampling frequency: 44.1 kHz, 16 bit). During the 3-minute isolation condition, a kitten was spatially separated from its mother and siblings. In the Low arousal condition a kitten was spatially isolated and left undisturbed on the floor of the animal room. In the High arousal condition a kitten was additionally handled by the experimenter (lifted off the ground and/or turned onto its back). After each condition, kittens were reunited with their mother and siblings, and mother and kittens resumed normal behaviours (e.g., nursing), without any signs of stress.

### Selection of kitten calls

**Fig. 1:**
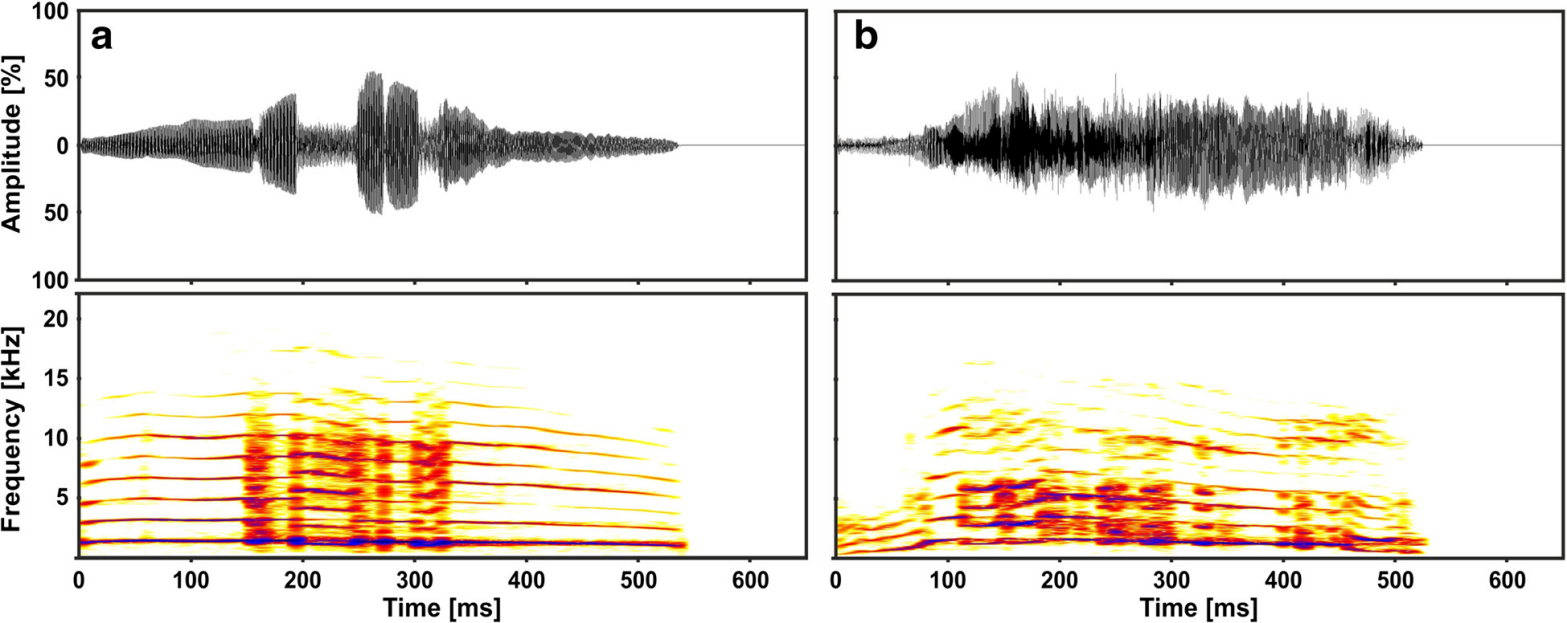
Oscillogram and Sonagram of Low and High arousal kitten calls. Depicted are kitten isolation calls of the same sender of the Low (**a**) and High (**b**) arousal condition

In our previous publication [11] we showed that kitten isolation calls of the two arousal conditions differ in call duration and spectral parameters related to the fundamental frequency. Pilot playback experiments showed that cats responded to playbacks of kitten calls within the average duration of a call. Thus, for the selection of playback stimuli (Fig. 1), we decided to use single calls instead of call series and chose continuous calls, with a call duration of approximately 630 ms to make sure that adult cats heard a similar proportion of the call (whether High or Low arousal), prior to responding. During the selection process, we excluded calls of low signal-to-noise ratio and took care that each subject was familiar with (e.g., mother or sibling of) no more than 2 kitten senders. Thus, we chose a set of 14 representative calls from 7 kittens (4 males, 3 females): One Low and one High arousal call from each sender. All 14 playback stimuli were used for all subjects. Thus, a subject could not use individual differences of kitten calls to discriminate the arousal conditions.

For the acoustic characterization of the selected playback stimuli an acoustic analysis using PRAAT (www.praat.org; [51]; see also Additional file 1) was performed. Based on previous results [11], we focused on temporal and spectral aspects of F0. Measurements of F0 were performed extracting the F0 contour using the To Pitch (cc) command in Praat (time steps: 0.005 s; pitch floor: 500 Hz; pitch ceiling: 3000 Hz). We used the pitch target segment to compare the extracted pitch contour with the sonagram and corrected the data if necessary. The following 10 acoustic parameters were obtained (Table 1): duration – time between on- and offset of the call; minF0 – minimum F0 of the call; timeminF0 – time between onset and minF0; maxF0 – maximum F0 of the call; timemaxF0 – time between onset and maxF0, F0 range – difference between minF0 and maxF0; meanF0 – mean value of F0 throughout the call; sDF0 – standard deviation of F0 throughout the call; F0start – fundamental frequency (F0) at the start of the call (first 5-ms time-frame); F0slope - slope from startF0 to maxF0, calculated in octaves (log (maxF0 – F0start)/ log(2)), as octaves/timemaxF0. The stimuli were distinct with regard to the arousal state, with Low and High arousal calls differing significantly in minF0 and sDF0 (paired t-tests: *p* ≤ 0.033; F0start: *p* = 0.054; Fisher Omnibus test: χ^2^ = 44.7, df = 20, *p* = 0.001).

**Table 1:** Acoustic properties of kitten call playback-stimuli: 7 Low and 7 High arousal calls

	Low arousal (mean + SD)	High arousal (mean + SD)
Duration [ms]	594 + 77	659 + 112
minF0 [Hz]	1134 + 320	756 + 184
timeminF0 [ms]	334 + 312	124 + 290
maxF0 [Hz]	1699 + 230	1593 + 262
timemaxF0 [ms]	221 + 38	266 + 95
F0 range [Hz]	565 + 167	837 + 281
meanF0 [Hz]	1468 + 269	1316 + 159
sDF0 [Hz]	127 + 29	212 + 83
F0start [Hz]	1241 + 396	850 + 286
F0slope [octave/s]	2.3 + 1.5	3.9 + 2.0

### Processing of playback stimuli

Kitten calls (sampling frequency: 44,100 Hz) were cut at zero-crossings of the oscillogram (Signal 4.0, Engineering Design, Berkeley, CA, USA), individually high-pass filtered and low-pass filtered at 20,000 Hz (BatSound Pro, Pettersson Elektronik AB, Uppsala, Sweden). Each stimulus was equipped with a short sequence of silence (0.2 ms, Signal 4.0) at the beginning of the call, to eliminate onset clicks and was prolonged to 3 s total duration by adding silence (Signal 4.0). All stimuli were played back at 70 ± 2 dB sound pressure level (RMS fast measurement: Bruel and Kjær 2610, high-pass filter: 22.4 Hz), at hearing distance during the experiments (see below), to match the loudness of natural kitten vocalisations [45].

### Playback experiments and experimental set-up

**Fig. 2:**
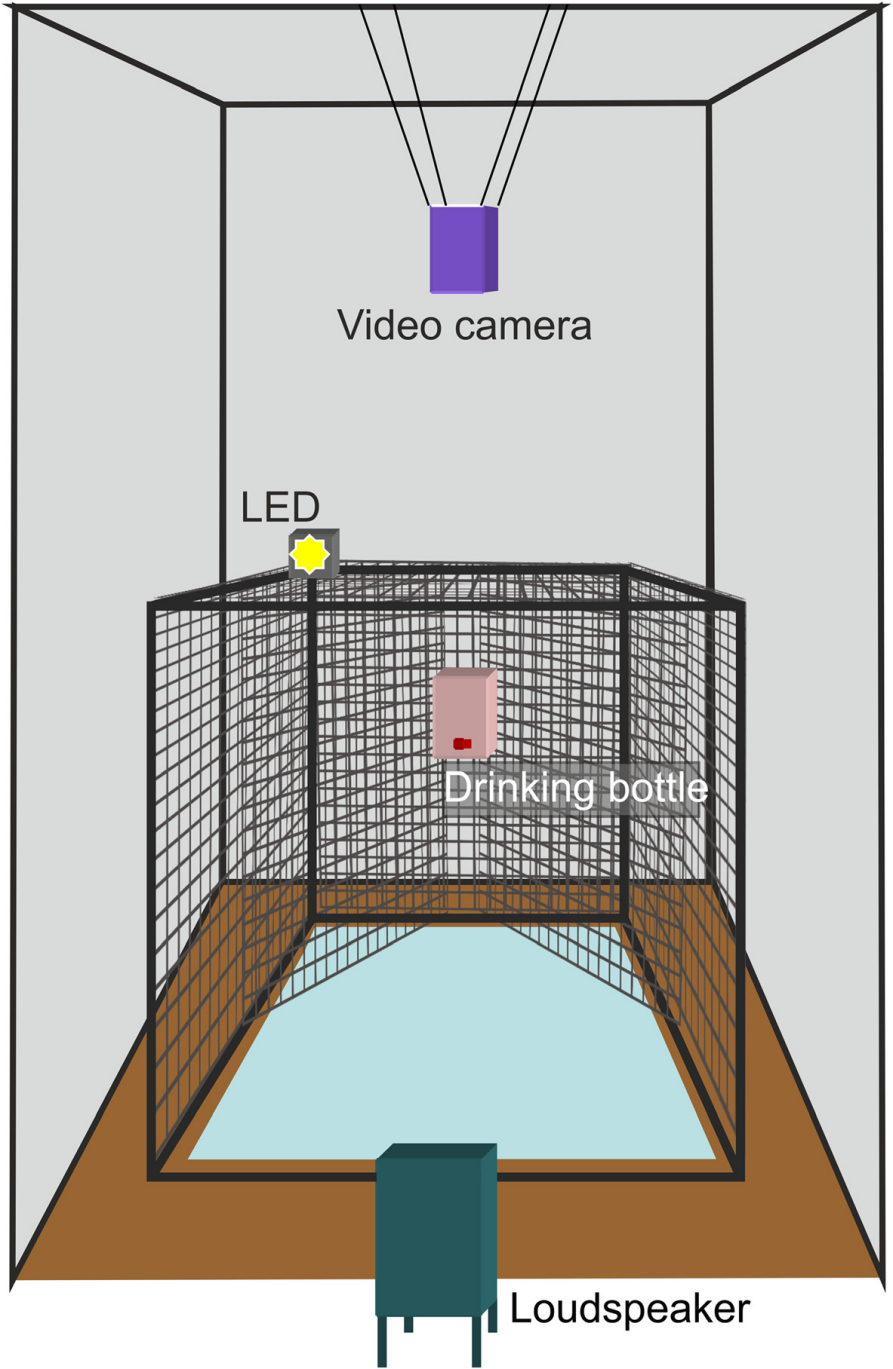
Experimental set-up

The cats were tested individually in a separate testing room. In the centre of the testing room an experimental cage (wire dog crate, 54 x 78 x 62 cm^3^) was placed on a carpet, surrounded by sound attenuating foam, attached to 4 movable walls (Fig. 2). The loudspeaker (quadral Argentum 02.1, quadral GmbH & Co. KG) was placed behind an opening in the foam of one movable wall. Opposite to the loudspeaker, the experimental cage was equipped with a drinking bottle containing a milk/water solution. The cage was equipped with wire mesh to guarantee that the subjects were aligned closely to the bottle-loudspeaker axis while drinking. The experiments were performed and monitored from outside the testing room via an observational camera and a laptop. Video samples were recorded with a digital camera (Sony DCR-SR75E, Tokyo, Japan) suspended over the bottle site. The two camera signals were synchronised to the playback presentations via a diode light, indicating the duration of sound presentation. The light was visible to both cameras, but invisible to the subjects. The playback stimuli were played back via a Marantz recorder (PMD 671) and an HK 980 amplifier (harman/kardon, HARMAN International Industries, Inc., Stamford, CT, USA).

Before playback experiments started, we habituated each subject to the experimental setup and the experimental procedure within 5- to 10-min trials, 2–4 times a week. We defined a subject as habituated if it showed no signs of stress (e.g., escape attempts from the cage, or intensive vocalisation) and was drinking from the bottle for at least 10 s during 5 min. When a subject reached the habituation criterion, we conducted the first playback experiment the next session.

A playback experiment started half a minute after the observer had left the testing room. The stimuli were presented in a pseudo-randomised order, with one stimulus being played no more than twice consecutively. Stimuli were played back only when the subject was licking the drinking bottle. To ensure independent behavioural responses, subsequent playback presentations were played at intervals of at least 1 min. To reduce the impact of varying motivational states throughout a session (i.e., motivation to drink), behavioural responses were recorded over several sessions with no more than 4 stimuli being played during each session and a session being stopped at the latest after 15 min. The cage was cleaned with disinfectant after each experiment and the two sexes were tested in two different cages, identical in construction. Each cat was tested 2 to 4 times a week. The experiment of one subject was completed when each of the 14 different stimuli had been scored in the video analysis.

### Video analyses

Video analysis was performed blind to the respective playback stimulus (i.e., without acoustic information) using the visual cue of the diode. A stimulus presentation was analysed only when a subject had contact to the drinking bottle at the first flashing of the diode.

We scored all behaviours occurring within a defined time-frame after stimulus onset (see below): Stop drinking (without turning the head or body), partial head turn (less than 180°), partial body turn (forelimbs were moved in direction of the head turn) and orientation to the loudspeaker (head or body turn with gaze oriented to the loudspeaker). Vocalisations and marking behaviour only occurred in a subset of individuals (males and females) and were therefore excluded from further analyses. As orientation to the loudspeaker was the most frequent response (cf. results), we chose its onset latency as a measure of responsiveness. For this behaviour, we analysed the first second after stimulus onset in slow motion (replay speed: 14 frames/s) with Interact 32 software (Version 8, Mangold, Arnstorf, Germany) and scored latencies with an accuracy of 0.04 s. The one-second time frame was defined via an analysis of the frequency-distribution of latencies (*n* = 85) over a total duration of 5 s, which showed that 85 % of the responses occurred in a time-frame of up to 1 s. Responses after 1 s were supposed to be random behaviours.

Inter-observer reliability was high; 25 % of the stimuli were reanalysed by a second observer (latency to orientate to the loudspeaker: Two-tailed Spearman-Rho correlation: *p* < 0.001, *r* = 0.99).

### Statistical analyses

Due to longitudinal repeated measurements [52] we used generalised estimating equation (GEE) models to assess the influence of the explanatory variables Sex (male/ female) and Arousal (high/ low) on the dependent variable response latency. The GEE approach fits marginal mean models considering “correlated observations within clusters without fully specifying the joint distribution of the observation” [53]. Due to a potential habituation effect, we expected correlations between within-subject observations (i.e., measurement at time s is dependent on the measurement at time s-1). Thus, we specified an auto-regressive correlation structure (Trial number) and used Subject as grouping variable [52]. The analysis was performed using R (R version 3.1.1 (2014-07-10); R Core Team, 2014) and the package ‘geepack’ [53–55]. First, a full GEE model was set up, with the main terms Sex and Arousal and the interaction term (Sex*Arousal), Subject as grouping variable and Trial number as within-subject factor, which was modelled using the AR-1 correlation [53]. We used a backward stepwise elimination procedure to determine the minimum adequate model (final model; [52]). Each time we dropped the highest-level interaction with the highest non-significant *p*-value and compared the previous to the reduced nested model using Wald test statistics ('anova' command; [53]). The elimination procedure was stopped when (1) the Wald test indicated a significant difference between the two models (the previous model was selected), or (2) only main terms remained in the final model. In the result section we only report on the final models. To explain significant interaction terms, we performed a break-down analysis by splitting our dataset. When evaluating only females, we included previous experience with kittens as an additional explanatory variable Experience (naïve/ experienced) in the GEE model. For the significant terms in the final model we also reported the odd ratio and its confidence interval to estimate the effect size of the explanatory variable.

To investigate whether temporal and spectral parameters characterizing F0 (cf. Table 1) correlated with response latency, we used a Pearson correlation using SPSS (SPSS 23, IBM). To control for multiple testing we applied the Fishers Omnibus test combining multiple *p*-values [56].

## Results

### Qualitative analysis of behavioural responses

The subjects responded to 61 % of the playback-presentations (*n* = 238): in 9 % they stopped drinking, in 15 % they showed a partial head turn, in less than 1 % they showed a partial body turn and in 36 % they turned directly to the loudspeaker. As orientation to the loudspeaker was the most frequent response, we chose its onset latency as a measure of responsiveness. The raw data for response latency is available in Additional file 1.

### Response latency towards playback stimuli

**Fig. 3:**
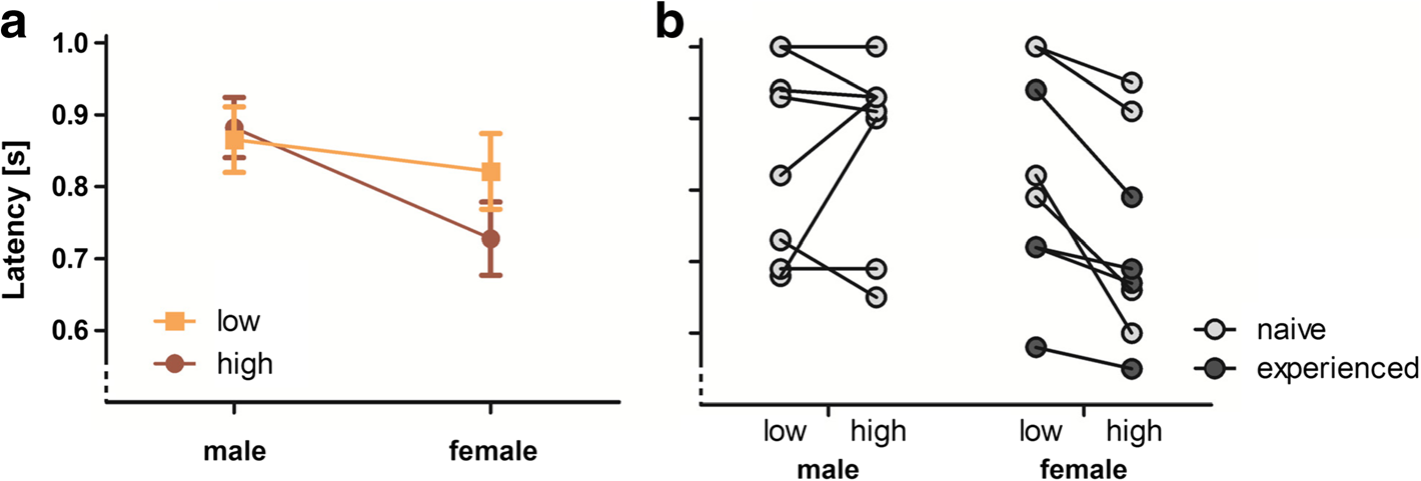
Response latency for each sex and arousal condition. Mean response latencies **a** showed a significant Sex*Arousal interaction (GEE model: *p* < 0.001). Given are means (symbol) and standard deviations (whisker). Individual responses **b** revealed that all females (naïve and experienced) responded faster to High compared to Low arousal calls. Individual data are connected by lines

The final model (Sex*Arousal) revealed a significant effect for the main term Arousal (W = 4.75, *p* = 0.029; odds ratio: 1.11; CI: 1.01-1.23) but not for Sex (W = 1.10, *p* = 0.295). However, a significant interaction between Arousal and Sex (W = 11.81, *p* < 0.001; odds ratio: 0.90; CI: 0.84-0.95) suggested that arousal conditions affect the response latency of males and females differently (Fig. 3). Thus, we conducted step-down analyses for males and females, separately.

Males showed a similar response latency towards calls of Low and High arousal condition (W = 0, *p* = 0.96). For female cats, we also included previous experience with kittens as additional main term (Arousal*Experience; Fig. 3b). After backward reduction, the final model revealed significant effects of the main term Arousal (W = 22.67, *p* < 0.001) but not for Experience (W = 1.97, *p* = 0.160). Thus, female cats responded about 10 % faster to playback stimuli of the High arousal than the Low arousal condition (odds ratio: 0.90; CI: 0.86-0.94).

### Correlation with acoustic parameters related to the fundamental frequency

**Fig. 4:**
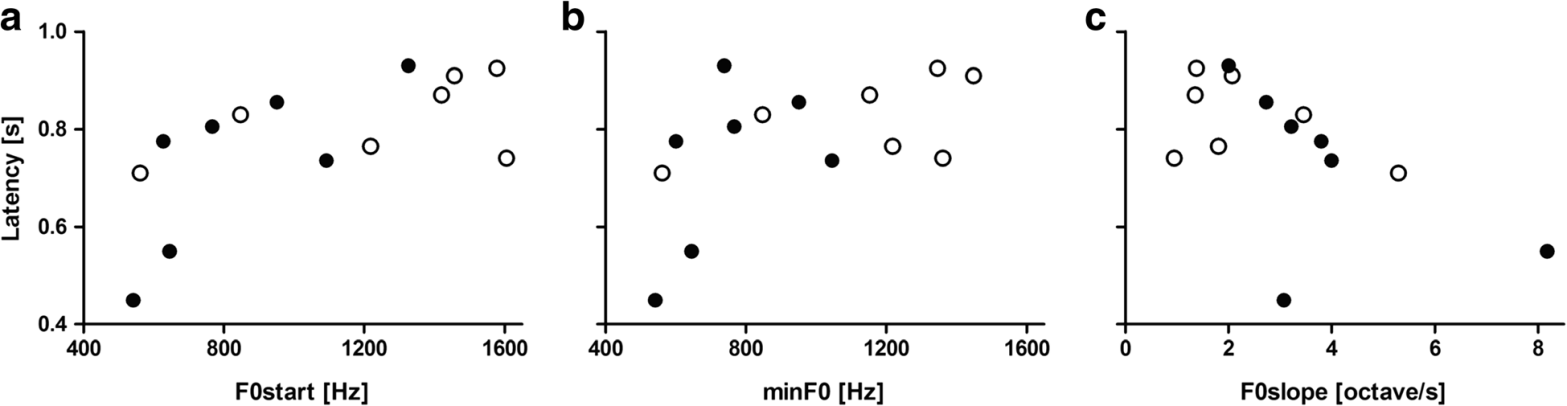
Correlation between female response latency and source-related acoustic parameters of kitten isolation calls. The response latency was significantly correlated (Pearson correlation: *p* ≤ 0.047) with F0start (**a**), minF0 (**b**) and F0slope (**c**). Dots represent means of all females (*N* = 8) for the Low (open) and High (filled) arousal kitten calls

Based on the finding that females responded faster to High than Low arousal calls, we assessed, whether the females response latency correlated with temporal and spectral parameters related to the F0 of kitten isolation calls (*N* = 14). We revealed a positive correlation between female response latency and F0start (*r* = 0.645, *p* = 0.013; Fig. 4a) as well as minF0 (*r* = 0.540, *p* = 0.047; Fig. 4b) and a negative correlation with the F0slope (*r* = -0.578, *p* = 0.031; Fig. 4c; Fisher Omnibus test: χ^2^ = 41.3, df = 20, *p* = 0.003) whereas the other parameters showed no significant correlation (duration: *r* = −0.285; timeminF0: *r* = 0.308; maxF0: *r* = 0.500; timemaxF0: *r* = −0.211; F0 range: *r* = −0.190; meanF0: *r* = 0.527; sDF0: *r* = -0.100; for all *p* ≥ 0.053).

## Discussion

In accordance with our hypothesis female cats adjusted their responsiveness towards the arousal conveyed by kitten calls, whereas males showed a similar responsiveness to kitten calls of both the Low and the High arousal condition. Thereby, female responsiveness correlated with changes in three source-related acoustic parameters (F0start, minF0 and F0slope). The present results indicate that differences in social environment due to a difference in parental investment and the resulting difference in behavioural relevance of species-specific vocalisations put distinct selective pressure on male and female cats, resulting in different auditory and/ or emotional processing.

The fact that female cats responded faster to kitten calls of the High versus the Low arousal condition corresponds well to previous results in other vertebrates (pigs: [39]; caimans: [40]), indicating that females possess the ability to adjust their motivation to respond adaptively to the emotional state of their young based on acoustic cues, only. In contrast, male cats did not adjust their responsiveness (i.e., latency to respond) based on the acoustic structure of the calls, which might be explained by the fact that infant calls are not behavioural relevant for males due to the absence of paternal care [49].

Females showed an elevated responsiveness to kitten calls with a lower F0start, lower minF0 and higher F0 slope. This finding corresponds to our previous result, demonstrating that kitten calls in the High arousal condition have a lower F0 than in the Low arousal condition. The elevated responsiveness to low frequencies (start and minF0) cannot be explained merely by hearing sensitivity, as the area of best hearing in cats is around 2 kHz [57], which is even higher than the maxF0 of the tested kitten isolation calls. However, lower frequencies at the start of the call (which were also most often the lowest values throughout the call, i.e., minF0), resulted in a steeper F0slope (octave/s), activating a higher proportion of the auditory pathway in a given time interval, due to an almost-exponential distribution of the cochlear map [58]. The dependency of maternal responsiveness on F0 is in agreement with studies in humans (e.g., [59–61]) and deer [5, 6]. Deer females respond more strongly to isolation calls presented in a preferred, species-specific frequency range [5].

From our female subjects, half had already raised own offspring previous to the experiment, whereas the other half were virgins without any experience with kittens. However, we found no difference between both groups, with all females responding faster to High than Low arousal kitten calls. This finding suggests that experience plays, if at all, only a limited role in the discrimination of arousal in infant calls. Thus, similar to findings in other species, including humans, already naïve/ non-parent female cats were able to evaluate the emotional content of infant vocalisation (humans: [35, 38, 62]; laboratory mice: [24, 25, 32]). We have to point out that, in order to compare males and females regardless of experience, we used calls from unrelated kittens. As kitten calls contain individual signatures [11], it can be hypothesised that cat mothers are able to learn the voices of their own kittens and further adapt to their developmental changes [46]. As being behaviourally more relevant, calls of their own kittens might result in pronounced response differences between Low and High arousal calls. Future studies shall address this point further and analyse whether this more specific experience might enhance the differentiation of arousal in kitten isolation calls, leading to a difference in responsiveness between mothers and naïve females.

Taken together, our results indicate that the ability to adjust responsiveness to emotional cues of kitten calls is an ingrained (adult) sex difference, which does not need to be triggered by experience. These sex differences may be perceptual (i.e., auditory processing) or motivational (i.e., emotional processing) or a combination of the two. Sex differences in the auditory system of the domestic cat have not been described so far. However, more research is necessary to assess potential sexual dimorphic anatomical or functional characteristics of the auditory system in domestic cats.

## Conclusion

We assessed for the first time whether in domestic cats, a species without paternal care, both males and females adjust their responsiveness to the voice of kittens. We revealed a sex-specific responsiveness to kitten isolation calls recorded in different arousal conditions. Thereby, females, but not males, adjusted their responsiveness according to the conveyed urgency to respond. This sex-difference can be explained by the absence of paternal care. Experience with kittens was not necessary for arousal-specific responsiveness, as also naïve females responded stronger to High than Low arousal calls. These changes in female responsiveness correlated with changes in spectral parameters of the fundamental frequency of kitten calls. We propose that the maternal breeding system has shaped auditory and/or emotional processing distinctively in female compared to male cats.

## Abbreviations

F0 range, difference between minF0 and maxF0; F0, fundamental frequency; F0slope, slope from startF0 to maxF0 as octaves/timemaxF0; F0start, fundamental frequency at call onset; GEE, Generalised estimating equation; maxF0, maximum F0 of the call; meanF0, mean value of F0 throughout the call; minF0, minimum F0 of the call; sDF0, standard deviation of F0 throughout the call; timemaxF0, time between onset and maxF0; timeminF0, time between onset and minF0

## Additional file

Additional file 1 Raw data for GEE and acoustic analyses. (XLSX 18 kb)

## References

[1] Zeifman DM (2001). An ethological analysis of human infant crying: answering Tinbergen's four questions. Dev Psychobiol.

[2] Lingle S, Wyman MT, Kotrba R, Teichroeb LJ, Romanow CA (2012). What makes a cry a cry? A review of infant distress vocalizations. Curr Zool.

[3] Chang RS, Thompson NS (2011). Whines, cries, and motherese: their relative power to distract. J Soc Evol Cult Psychol.

[4] Ehret G (2005). Infant rodent ultrasounds – a gate to the understanding of sound communication. Behav Genet.

[5] Lingle S, Riede T (2014). Deer mothers are sensitive to infant distress vocalizations of diverse mammalian species. Am Nat.

[6] Teichroeb LJ, Riede T, Kotrba R, Lingle S (2013). Fundamental frequency is key to response of female deer to juvenile distress calls. Behav Process.

[7] Wood RM (2009). Changes in cry acoustics and distress ratings while the infant is crying. Infant Child Dev.

[8] Scheumann M, Zimmermann E, Deichsel G (2007). Context-specific calls signal infants' needs in a strepsirrhine primate, the gray mouse lemur (*Microcebus murinus*). Dev Psychobiol.

[9] Thomas TJ, Weary DM, Appleby MC (2001). Newborn and 5-week-old calves vocalize in response to milk deprivation. Appl Anim Behav Sci.

[10] Stoeger AS, Charlton BD, Kratochvil H, Fitch WT (2011). Vocal cues indicate level of arousal in infant African elephant roars. J Acoust Soc Am.

[11] Scheumann M, Roser AE, Konerding W, Bleich E, Hedrich HJ, Zimmermann E (2012). Vocal correlates of sender-identity and arousal in the isolation calls of domestic kitten (*Felis silvestris catus*). Front Zool.

[12] Stoeger AS, Baotic A, Li DS, Charlton BD (2012). Acoustic features indicate arousal in infant giant panda vocalisations. Ethology.

[13] Camaclang AE, Hollis L, Barclay RMR (2006). Variation in body temperature and isolation calls of juvenile big brown bats, *Eptesicus fuscus*. Anim Behav.

[14] Illmann G, Schrader L, Spinka M, Sustr P (2002). Acoustical mother-offspring recognition in pigs (*Sus scrofa domestica*). Behaviour.

[15] Briefer E, McElligott AG (2011). Mutual mother-offspring vocal recognition in an ungulate hider species (*Capra hircus*). Anim Cogn.

[16] Insley JI, Phillips AV, Charrier I (2003). A review of social recognition in pinnipeds. Aquat Mamm.

[17] Haskins R (1977). Effect of kitten vocalizations on maternal behavior. J Comp Physiol Psych.

[18] Shizawa Y, Nakamichi M, Hinobayashi T, Minami T (2005). Playback experiment to test maternal responses of Japanese macaques (*Macaca fuscata*) to their own infant's call when the infants were four to six months old. Behav Process.

[19] Simons RC, Bobbitt RA, Jensen GD (1968). Mother monkeys (*Macaca nemestrina*) responses to infant vocalizations. Percept Mot Skills.

[20] Balcombe JP (1990). Vocal recognition of pups by mother Mexican free-tailed bats, *Tadarida brasiliensis mexicana*. Anim Behav.

[21] Bohn KM, Wilkinson GS, Moss CF (2007). Discrimination of infant isolation calls by female greater spear-nosed bats, *Phyllostomus hastatus*. Anim Behav.

[22] Ehret G, Koch M (1989). Ultrasound‐induced parental behaviour in house mice is controlled by female sex hormones and parental experience. Ethology.

[23] Hahn ME, Lavooy MJ (2005). A review of the methods of studies on infant ultrasound production and maternal retrieval in small rodents. Behav Genet.

[24] Smith JC (1976). Responses of adult mice to models of infant calls. J Comp Physiol Psych.

[25] Okabe S, Nagasawa M, Kihara T, Kato M, Harada T, Koshida N, Mogi K, Kikusui T (2010). The effects of social experience and gonadal hormones on retrieving behavior of mice and their responses to pup ultrasonic vocalizations. Zool Sci.

[26] Zahed S, Prudom S, Snowdon C, Ziegler T (2008). Male parenting and response to infant stimuli in the common marmoset (*Callithrix jacchus*). Am J Primatol.

[27] Barbosa MN, da Silva Mota MT (2014). Do newborn vocalizations affect the behavioral and hormonal responses of nonreproductive male common marmosets (*Callithrix jacchus*)?. Primates.

[28] Sánchez SM, Ziegler TE, Snowdon CT (2014). Both parents respond equally to infant cues in the cooperatively breeding common marmoset, *Callithrix jacchus*. Anim Behav.

[29] Lingle S, Rendall D, Wilson WF, Deyoung RW, Pellis SM (2007). Altruism and recognition in the antipredator defence of deer: 2. Why mule deer help nonoffspring fawns. Anim Behav.

[30] Ziegler TE, Jacoris S, Snowdon CT (2004). Sexual communication between breeding male and female cotton-top tamarins (*Saguinus oedipus*), and its relationship to infant care. Am J Primatol.

[31] Fernandez-Duque E, Valeggia CR, Mendoza SP (2009). The biology of paternal care in human and nonhuman primates. Annu Rev Anthropol.

[32] Ehret G, Koch M, Haack B, Markl H (1987). Sex and parental experience determine the onset of an instinctive behavior in mice. Die Naturwissenschaften.

[33] Ehret G (1988). Physiologische Grundlagen der Entwicklung akustischer Kommunikation bei Säugetieren. Verh Deut Z.

[34] Gustafson GE, Green JA (1989). On the importance of fundamental frequency and other acoustic features in cry perception and infant development. Child Dev.

[35] Seifritz E, Esposito F, Neuhoff JG, Lüthi A, Mustovic H, Dammann G, Von Bardeleben U, Radue EW, Cirillo S, Tedeschi G (2003). Differential sex-independent amygdala response to infant crying and laughing in parents versus nonparents. Biol Psychiatry.

[36] Sander K, Frome Y, Scheich H (2007). FMRI activations of amygdala, cingulate cortex, and auditory cortex by infant laughing and crying. Hum Brain Mapp.

[37] Scheumann M, Hasting AS, Kotz SA, Zimmermann E (2014). The voice of emotion across species: how do human listeners recognize animals' affective states?. PLoS One.

[38] Lindová J, Špinka M, Nováková L (2015). Decoding of baby calls: can adult humans identify the eliciting situation from emotional vocalizations of preverbal infants?. PLoS One.

[39] Weary DM, Lawson GL, Thompson BK (1996). Sows show stronger responses to isolation calls of piglets associated with greater levels of piglet need. Anim Behav.

[40] Vergne AL, Aubin T, Taylor P, Mathevon N (2011). Acoustic signals of baby black caimans. Zoology.

[41] Heid S, Hartmann R, Klinke R (1998). A model for prelingual deafness, the congenitally deaf white cat - population statistics and degenerative changes. Hear Res.

[42] Kral A, Hartmann R, Tillein J, Heid S, Klinke R (2002). Hearing after congenital deafness: central auditory plasticity and sensory deprivation. Cereb Cortex.

[43] Moelk M (1944). Vocalizing in the house-cat; a phonetic and functional study. Am J Psychol.

[44] Brown KA, Buchwald JS, Johnson JR, Mikolich DJ (1978). Vocalization in the cat and kitten. Dev Psychobiol.

[45] Romand R, Ehret G (1984). Development of sound production in normal, isolated and deafened kittens during the first postnatal months. Dev Psychobiol.

[46] Hubka P, Konerding W, Kral A (2015). Auditory feedback modulates development of kitten vocalizations. Cell Tissue Res.

[47] Härtel R (1975). Zur Struktur und Funktion akustischer Signale im Pflegesystem der Hauskatze (*Felis catus* L.). Biol Zbl.

[48] Kiley-Worthington M (1984). Animal language? Vocal communication of some ungulates, canids and felids. Acta Zool Fennica.

[49] Deag JM, Manning A, Lawrence CE, Turner DC, Bateson P (2000). Factors influencing mother-kitten relationship. The domestic cat: the biology of its behaviour.

[50] Haskins R (1979). A causal analysis of kitten vocalization: an observational and experimental study. Anim Behav.

[51] Boersma P (1993). Accurate short-term analysis of the fundamental frequency and the harmonics-to-noise ratio of a sampled sound. IFA Proceedings.

[52] Zuur AF, Ieno EN, Walker NJ, Saveliev AA, Smith GM (2009). Mixed effects models and extensions in ecology with R.

[53] Højsgaard S, Halekoh U, Yan J (2006). The R package geepack for generalized estimating equations. J Stat Softw.

[54] Yan J (2002). Geepack: yet another package for generalized estimating equations. R-News.

[55] Yan J, Fine J (2004). Estimating equations for association structures. Stat Med.

[56] Haccou P, Melis E (1994). Statistical analysis of behavioural data.

[57] Sokolovski A (1973). Normal threshold of hearing for cat for free-field listening. Arch Klin Exp Ohr.

[58] Greenwood DD, Joris PX (1996). Mechanical and ''temporal'' filtering as codeterminants of the response by cat primary fibers to amplitude-modulated signals. J Acoust Soc Am.

[59] Zeskind PS, Marshall TR (1988). The relation between variations in pitch and maternal perceptions of infant crying. Child Dev.

[60] Zeskind PS, Shingler EA (1991). Child-abusers perceptual responses to newborn-infant cries varying in pitch. Infant Behav Dev.

[61] Dessureau BK, Kurowski CO, Thompson NS (1998). A reassessment of the role of pitch and duration in adults' responses to infant crying. Infant Behav Dev.

[62] Zeskind PS, Sale J, Maio ML, Huntington L, Weiseman JR (1985). Adult perceptions of pain and hunger cries: a synchrony of arousal. Child Dev.

